# Chain Formation and Addition Drive the Debye Relaxation of Methanol

**DOI:** 10.1021/acs.jpcb.5c04122

**Published:** 2025-08-25

**Authors:** Rebecca A. Bone, Moses K. J. Chung, Jay W. Ponder, Kathleen Schwarz

**Affiliations:** † 528984 Theiss Research, P.O. Box 127, La Jolla, California 92038, United States; ‡ Material Measurement Laboratory, 10833 National Institute of Standards and Technology, 100 Bureau Dr., Gaithersburg, Maryland 20899, United States; § Department of Chemistry, 12275 Washington University in St. Louis, St. Louis, Missouri 63130, United States

## Abstract

The Debye relaxation in alcohols has generally been attributed to “collective motion” driven by hydrogen bonding, but the molecular mechanism of relaxation has yet to be conclusively identified. Taking methanol as a model alcohol, we apply an oscillating electric field in molecular dynamics simulations to directly evaluate the molecular motions and identify the mechanism. Methanol forms short-lived chains of hydrogen-bonded molecules through the hydroxyl group. We find that the hydroxyl group rotates in response to the field with a frequency dependence that tracks the Debye peak, implicating these chains in the Debye relaxation. Focusing on the hydroxyl OH rotation, we consider the events (e.g., diffusion of chains, birth, growth) over the lifetime of chains that could contribute to this frequency dependence. We find that molecular participation in chains is responsible for the OH alignment relative to the field: molecules align incrementally, ″clicking in″ during chain formation and during molecular addition to existing chains.

## Graphical abstract

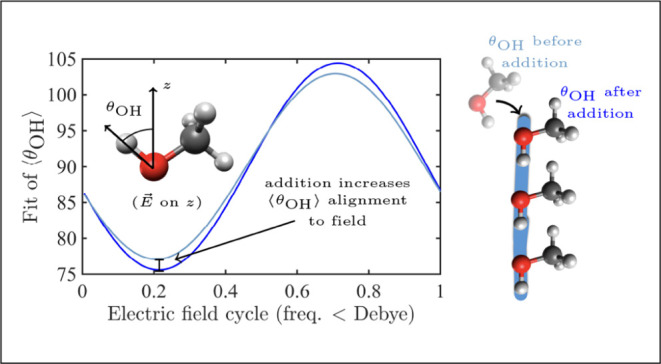

## Introduction

Hydrogen-bonding solvents are ubiquitous in nature and in industry, driving diverse processes from protein folding^1,2^ and proton transfer^3,4^ to hydrogen generation.^5,6^ Hydrogen-bonding solvents generally have large static dielectric constants, primarily due to the large magnitude of the Debye peak, the lowest frequency feature in their dielectric spectrum.^7–14^ Originally, Debye proposed that this peak originates from molecules individually responding to a field.^15,16^ However, this theory and other mean-field models have been found to apply only to limited situations^16^ certainly not including the collective relaxation of pure hydrogen-bonding solvents. Recently, observations of water^17–21^ suggest that this peak is instead attributable to bursts of collective reorientation of water’s hydrogen-bonding network.

In contrast, alcohols do not form networks, instead forming transient chains of hydrogen-bonded molecules.^22–25^ Focusing on the alcohol Debye relaxation, the transient chain model^25–28^ is the predominant model among many^29–31^ used to explain the collective reorientation in alcohols. In the transient chain model, many short-lived chains of hydrogen-bonded molecules constantly form, grow, shrink, and die. The Debye mechanism in the transient chain model is argued to be (1) chains form and grow (shrink and die) when aligned (unaligned) with the field^32^ (2) chains turn by growing—the “turn and grow” hypothesis—in the direction of the field,^22^ or (3) dissociation of molecules from chains.^33^ However, some argue that the correlation of the time scale of these interactions with the time scale of the Debye peak is incidental rather than causal.^34^ This last proposal is often associated with the mechanism proposed by Johari and Dannhauser^35,36^ for the α peak (the next lowest frequency peak in the spectrum,^37,38^ in which lone molecules reorient to the field and hydrogen-bonded molecules do not. Others argue that a string of hydrogen-bonded molecules will undergo an end-to-end reorientation via OH flipping,^23,39,40^

Here, we directly test these hypotheses by applying an oscillating electric field and observing the dielectric response of simulated molecules.^43^ By doing this, the molecular motions at a particular frequency can be analyzed rather than observing all motions at the same time. Additionally, observing the solvent with and without the field allows us to form a causal link between the field and the observed molecular motions. This allows us to deconvolute the various motions of molecules and assign mechanisms to spectral features. We use this method to investigate the mechanism of response of the Debye peak in methanol, the smallest primary linear alcohol. After eliminating the other possible mechanisms, we conclude that participation in chains drives molecular alignment to the field: molecules “click in” to chains during both chain formation and molecular addition to existing chains.

## Computational Methods

### Simulation Details

We simulate methanol using the atomic multipole optimized energetics for biomolecular applications (AMOEBA09) force field.^44^ Standard parameters for the AMOEBA09 force field are used, including an Ewald cutoff of 7.0 Å, a van der Waals cutoff of 9.0 Å, Halgren (HHG) mixing rules, and mutual polarization.

Because we use a polarizable force field, the polarization includes both static (time-invariant) and induced (time-variant) components. The static component includes the atom’s point charge *q*_*i*_ and its static dipole $\mu_{i}^{sta}$ . The induced component of the dipole on each atom $\mu_{i}^{ind}$ is determined from interactions with nearby atoms and with the applied electric field (if present). These polarization components P_α_, where α indicates x, y, or z, constitute the total box polarization evaluated at each time step:

$$P_{\alpha }\left(t\right)=\overset{N}{\underset{i}{\sum }}\left[q_{i}\alpha_{i}\left(t\right)+\mu_{i\alpha }^{sta}+\mu_{i\alpha }^{ind}\left(t\right)\right]$$ 1

We generate initial configurations of 500 molecules using packmol.^45^ Periodic simulation boxes are equilibrated for 2 ns with a time step of 1 fs in the isothermal–isobaric ensemble (NPT) at 1 atm and 300 K using a Nosé-Hoover thermostat and barostat and the Verlet integration algorithm. A 50 ns simulation is then conducted to collect polarization to compute the dielectric spectrum using the Fluctuation Dissipation Theorem (FDT) method in ref 41.

### In-Field Simulations

Following the direct electric field approach^43^ we apply a sinusoidal electric field^46^ in the *z*-direction with an applied field strength of *E*_0_ = 0.01 *V*/*Å* across our entire periodic simulation box at frequencies near the Debye peak. We previously verified for other solvents that this field strength produces spectra with the direct electric field approach that are within the linear response regime and agree with those collected using the FDT method (i.e., with no applied field).^43^ Additionally, for methanol, we verified agreement between FDT results and those from the direct field method for a set of frequencies, with results shown in Figure S2. We collect the polarization and position of every atom at a rate of 1000 frames per cycle. We then calculate various metrics (e.g., atomic polarization) for specific atoms, molecules, or groups of molecules at each time frame. At each applied field frequency, we simulate 1000 cycles and output the simulation box configurations at 1000 evenly spaced times per cycle. Thus, for a simulation at 10 GHz, 100 ns in total is simulated, with printing every 0.1 ps for a total of 1000 cycles of the field. At frequencies below 10 GHz, 100 ns are simulated, and values are averaged over the number of cycles occurring within that time.

### Averaging over cycles

We calculate a number of metrics in our analysis for each molecule and at each time frame. When we average over cycles, we average the metric values for the *i* ^th^ frame of each cycle together. This produces a time series of 1000 average values representing the variation in the metric across an average cycle of the field.

We then fit this average cycle time series to the equation:

2 $$\langle \theta \rangle_{\mathrm{cyc}}=A\sin\left(\omega t\right)+B\cos\left(\omega t\right)$$

Because the sign of the coefficient depends on the definition of the bond vector (OH vs HO), to simplify, we report the absolute values of parameters *A* and *B*.

## Results and Discussion

### Tracking bond orientations

Before we can investigate the collective reorientation, we need to know which part(s) of the molecule respond at the Debye peak frequency. To answer this, we apply an electric field at various frequencies in our molecular dynamics simulations to simulate the dielectric response^43^ and then track the molecular response. Experimental spectra^42,47–49^ and the resulting simulated dielectric response (following the method in ref 41) are in good agreement, as shown in Figure 1c, within an offset from the overestimated simulated static dielectric constant (see Supporting Information).

**Figure 1:**
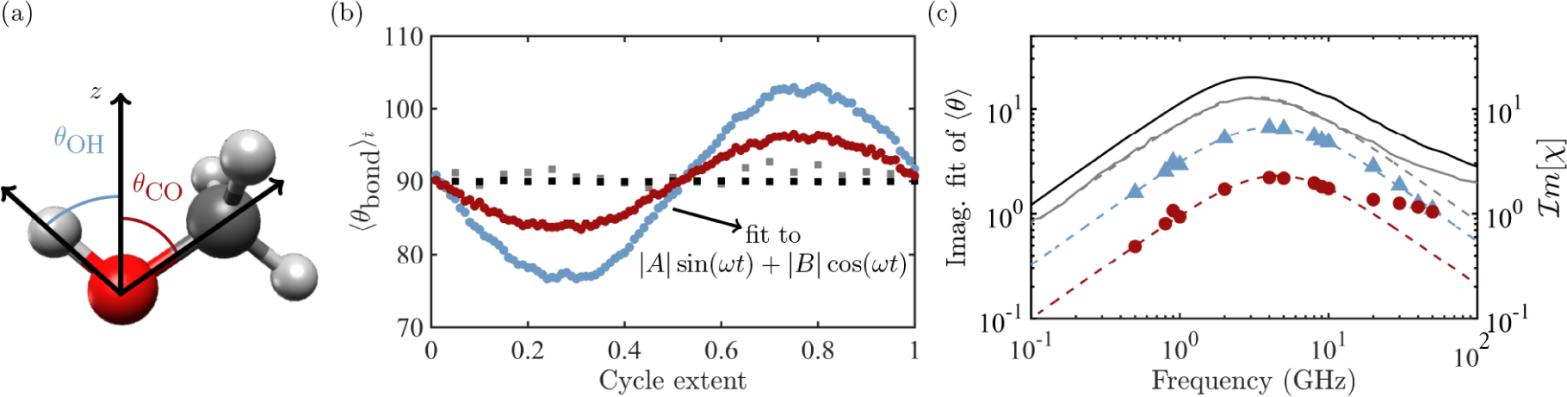
(a) Diagram of OH (blue) and CO (red) bond angle to *z*-axis, the direction of the applied field. (b) Average per-molecule OH (blue) and CO (red) bond angles relative to the *z*-axis over an average cycle at 0.5 GHz, compared to those without an applied field for CO (black) and OH (gray) (averaged as if a 10 GHz field were applied). (c) Comparison of the imaginary fitting parameter *B* of the OH (blue markers) and CO (red markers) angles and their Debye fits (dashed lines) on the left *y*-axis to the imaginary components of the simulated (black line, calculated as in ref 41) and experimental (gray line, ref 42 spectra on the right *y*-axis).

Tracking the molecular bond angles relative to the applied field (diagram in Figure 1a), we find that they vary sinusoidally with the field (shown for hydroxyl OH and CO angles in Figure 1b). Fitting to eq 2, we find that the |A| prefactor of the fitting for the hydroxyl OH bond tracks the real component of the Debye peak and, similarly, that the |B| prefactor tracks the imaginary component of the Debye peak (Figure 1c). Thus, the hydroxyl group rotates to align with the field over the course of a cycle. This implicates a reorientation of the hydroxyl group as the mechanism of the Debye peak. Further, the CO bond angle also responds in the frequency range of the Debye peak but with a considerably lower magnitude of response than the OH. Considering the fact that the oxygen atom is shared by both the CO and OH, this is evidence that the OH rather than the CO drives the response.

Lastly, we can differentiate these responses because the CO bond response has an additional feature on the high-frequency side of the Debye peak that is not present for the OH bond. This feature, known as the α relaxation (see, e.g., ref 37) is observed in experiments and in our calculated imaginary spectrum (gray and black solid lines in Figure 1). Thus, the OH bond is the primary driver of the Debye peak, and the CO bond is responsible for the α peak, in agreement with conclusions drawn from experimental findings.^22^ This allows us to deconvolute responses for these adjacent peaks with overlapping frequency ranges.

### Chains of hydrogen-bonded molecules

We now consider how methanol’s hydrogen bonding leads to chain structures in solution. A given pair of alcohol molecules is hydrogen bonded when they are close enough and their hydroxyl groups are aligned,^50^ as illustrated by the blue-highlighted hydroxyl groups in Figure 2 (see Supporting Information for a detailed algorithmic definition of hydrogen bonding in methanol). Chains of hydrogen-bonded molecules can be formed where one molecule is bound to another, which is bound to another, and so on. However, the oxygen atom’s two lone pairs can accept up to two hydrogen bonds, allowing each molecule to form up to three hydrogen bonds. The ability to create three hydrogen bonds means that these chains of hydrogen bonds can be branched to form more complicated arrangements of hydrogen-bonded molecules. On the other hand, not every molecule needs to be hydrogen-bonded. Such “lone” molecules can exist simultaneously with short and long chains that have varying amounts of branching.

**Figure 2:**
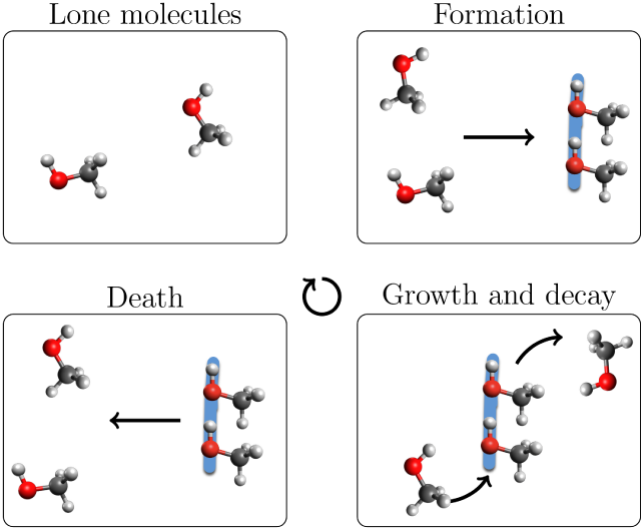
Life cycle of methanol chains.

We use the “life cycle” of a chain, Figure 2, to describe the exchange of molecules between the pool of lone molecules and those in chains. As shown in this figure, a chain first forms when two proximate lone molecules form a hydrogen bond. New molecules can add to or be removed from the chain, even to the point that no molecules are held in common from the beginning of the chain’s existence to the end. Eventually, the chain becomes a pair of hydrogen-bonded molecules that cease to be bound, becoming two lone molecules. This marks the “death” of the chain. During this time, other chains form, grow, decay, and die. At any given time, there are both lone molecules and chains of molecules to be tracked, each with a variety of histories, lengths, and structures. Algorithmic definitions of these events are provided in the Supporting Information.

### Lone molecules or chains?

The reorientation of the OH group could be undertaken via lone molecule rotational diffusion or chain dynamics (Figure 3). We first consider lone molecules, examining their lifetimes and their dipole moments. At any given time, lone molecules account for approximately 20% of all molecules in the simulation. However, the lifetime of lone molecules is exponentially distributed with a mean with an upper bound of 0.3 ps see Supporting Information for details), indicating that molecules are, on average, only very briefly unfettered by hydrogen bonds. The relatively short lifespan of lone molecules makes it unlikely that the Debye peak (whose time constant is approximately *τ_D_* = 51.5 ps) is attributable to lone molecules, though it does not preclude the idea.

**Figure 3:**
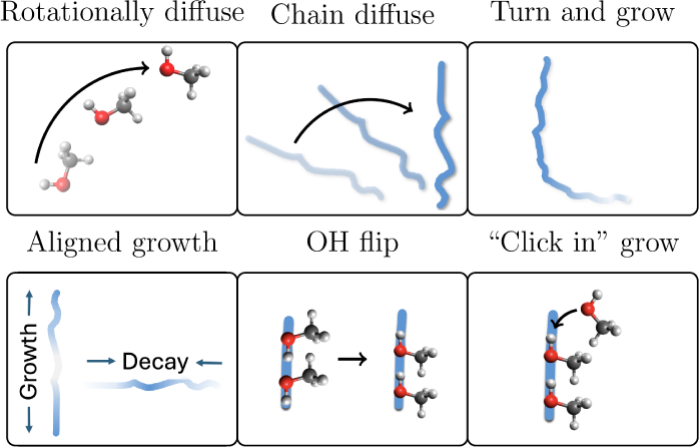
Possible hypotheses to explain the Debye relaxation of methanol, with the electric field pointed toward the top of the page.

Next, we compare the per-molecule polarization of lone molecules and hydrogen-bonded molecules. In this comparison, we consider the z-component, the direction of the applied field. The polarization of lone molecules varies sinusoidally with the field (Figure 4a), which is consistent with previous observations that lone molecules surrounding chains are themselves polarized.^34^ However, chained molecules are more polarized than lone molecules and have a greater OH bond angle response to the field (Figure 4b,c).

**Figure 4:**
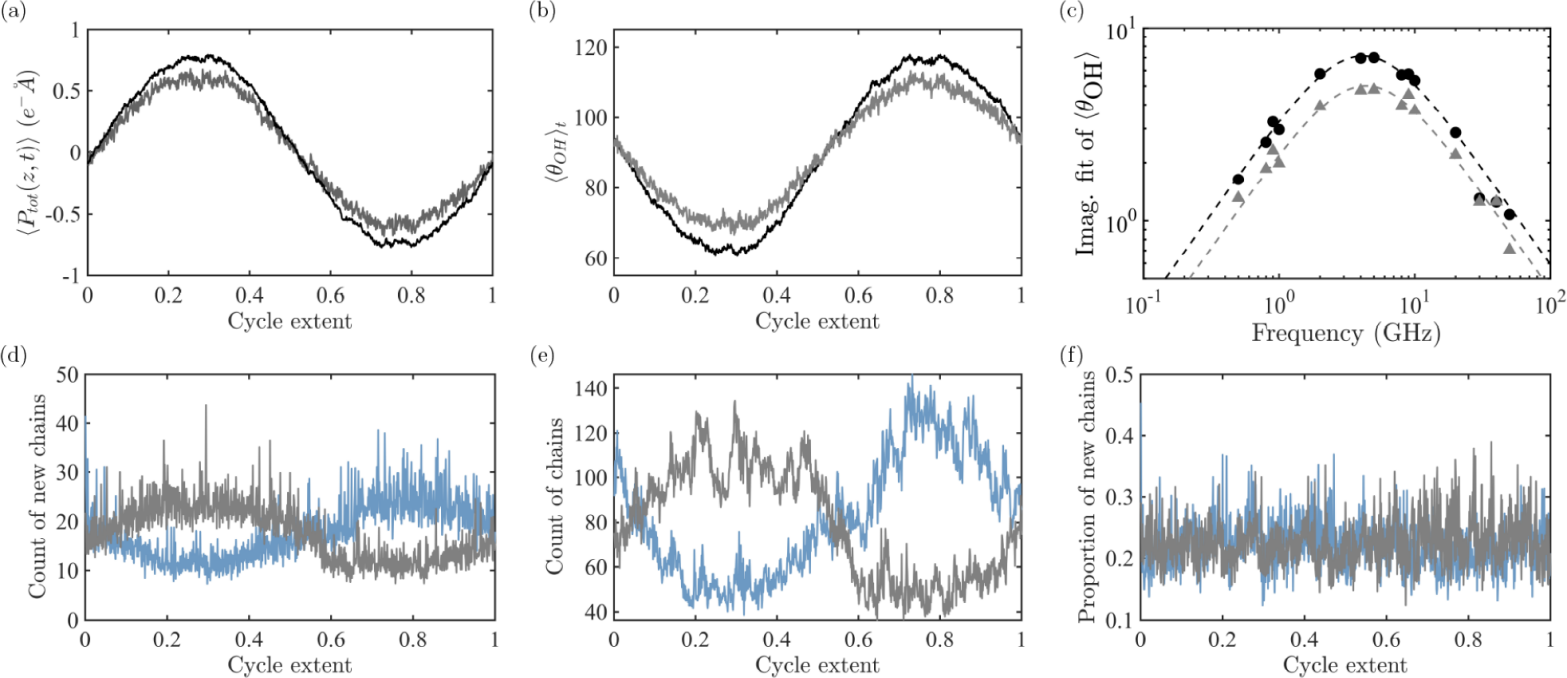
(a) Average z-component of the per-molecule polarization of lone molecules (gray) and chained molecules (black) across an average cycle at 0.5 GHz. (b) Average (per-molecule) OH bond angle relative to the *z*-axis of lone molecules (gray) and chained molecules (black) across an average cycle at 0.5 GHz. (c) Imaginary fitting parameter *B* of the OH bond angle relative to the *z*-axis of lone molecules (gray triangles) and chained molecules (black circles). (d–f) Average (d) count of new chains, (e) count of extant chains, and (f) proportion of new chains compared to all extant chains, separated into those whose average angle of the OH bond to the *z*-axis is greater than 90^°^ (blue) and less than 90^°^ (gray) at 1 GHz.

Additionally, we found that neither the dipole moment nor the OH angle relative to the field varies appreciably over the lifespan of lone molecules (Figure S9). We also found that the lifespan of chains is exponentially distributed, with a mean of 2.2 ps (Table S2). This is longer than the lifespan of lone molecules, yet shorter than the time constant associated with the Debye peak. This would indicate that not only does the alignment of the hydroxyl groups of chained molecules account for the Debye peak, but also that this alignment is incremental over the lifetime of many chains. Thus, we focus on the dynamics of the chains themselves.

### Investigating the life cycle of chains

The possible mechanisms that remain involve chains rather than lone molecules. We first consider hypotheses that require the OH angle to change over the lifetime of chains. Then, we will consider aligned growth and unaligned decay, ending with hypotheses that involve the addition and removal of molecules from the chain (including chain birth and death).

The hypotheses that require the OH angle to change over the lifetime of chains are: (1) chain diffusion, (2) turn and grow, and (3) OH flip. We observe that hydroxyl groups do not, on average, reorient over the lifetimes of individual chains (see Figure S9). Therefore, we can discount all three of these hypotheses.

To investigate aligned growth/unaligned decay, we separately count the forming and dying chains, and chains being added to and removed from, with OH angles greater and less than 90° at a frequency near but below the Debye peak, as shown in Figure 4(d–f). Both the above and below 90°counts vary sinusoidally with the field in each case (formation, death, addition, and removal). Facially, this would suggest that chains are preferentially formed in the direction of the field, grow aligned with the field, decay when unaligned with the field, and die when unaligned with the field. However, the counts alone are insufficient to prove this premise. Instead, we look at these counts in comparison to the total population of chains. When we do this, we find that the variation in these counts is reflective of the total population of chains. Take, for example, chain death. In order for chains to preferentially die when they are unaligned, the portion of chains dying that are unaligned must be greater than the portion of unaligned chains in the total chain population. We find that this is untrue for both chain birth and death. We can therefore eliminate the idea that chains are preferentially formed and grow in the direction of the field and decay and die when not aligned with the field.

Lastly, we consider events that occur in the life cycle of chains as the primary means of alignment. We collect the orientation of the hydroxyl group to the field just before (*A*_*i*_,*B*_i_) and just after (*A*_*f*_,*B*_*f*_) chain events and fit these responses separately using eq 2. We take the difference in these fitting parameters Δ*A* = |*A_f_* | – |*A_i_* |, as shown in Figure 5 (see Supporting Information for details, with individual parameters in Figure S2). When we consider the real response for each of these events, the 95% confidence intervals on the fit parameters before and after these events do not overlap at frequencies below the Debye peak. This indicates that there is a significant change in the OH orientation associated with each chain life event. Additionally, these changes are positive for chain formation and molecular addition and negative for chain death and molecular removal. This indicates that molecules align to the field by participating in chains, then lose that alignment when they leave chains.

**Figure 5:**
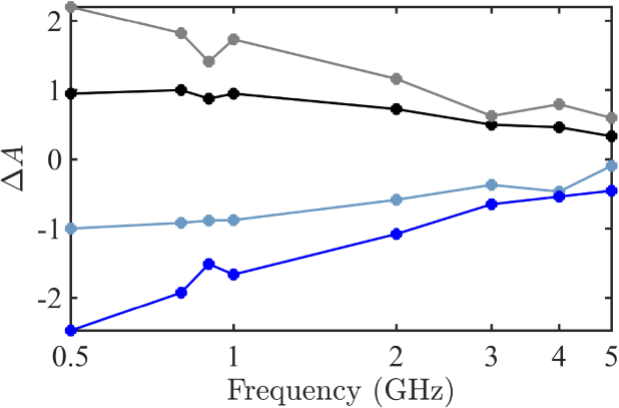
Change in the real response of the OH angle to the *z*-axis from addition to chains (gray), chain formation (black), chain death (light blue), and removal from chains (dark blue).

## Conclusions

In summary, we use molecular dynamics simulations to investigate the relaxation mechanism of the Debye peak in methanol. We observe short-lived branching chains whose underlying dynamics are consistent with the transient chain model. We apply an electric field at frequencies surrounding the Debye peak to directly observe the molecular motions responsible for this spectral feature. We find that the entire molecule rotates to respond to the field and that the reorientation of the hydroxyl group has the greatest angular magnitude of response. Further, the hydroxyl group does not have a response associated with the alpha peak, so we can leverage this to analyze the Debye peak separately from the alpha peak. The hydroxyl group’s primacy implicates the hydrogen bonding network (chains) in the mechanism of response of the Debye peak. We find that chain formation and molecular addition contribute to the alignment of molecules, and that chain death and molecular removal decrease the alignment of molecules: molecules “click in” and “click out”. This alignment persists up to the frequency of the Debye relaxation and decays following it.

Connecting this molecular response with chain dynamics, the mechanism of response first requires that chains exist and are born, grow, decay, and die. In the presence of a field, chains form on average slightly more aligned to the field than the two molecules were when they were alone. Chains grow approximately in this initial direction and do not appreciably diffuse or grow into a new direction. When molecules are added, the alignment of these molecules increases. When molecules are removed from chains, their alignment is reduced, but they do not continue to lose alignment through rotational diffusion. Instead, they quickly add to another chain. Thus, participation in chains incrementally increases the alignment of molecules at time scales associated with the Debye peak, linking the extensive observations of chains in alcohols with their relaxation behavior. We anticipate that this mechanism will straight-forwardly apply to explain the Debye peak of the other primary linear monoalcohols, with the caveat that longer alkyl groups may change the lifetimes and branching rates of the chains. This leaves for future research the generality of this mechanism for other hydrogen-bonding liquids.

## Supplementary Material



## Notes

Detailed methods (code, spectral calculation and fitting, benchmark dielectric spectrum, querying chain events, angle between specified bond and applied field, hydrogen bond identification, algorithm for tracking chains of molecules in time, dipole moment of hydrogen bound molecules, and additional MD analysis (PDF)

## References

[1] Pace C. N., Fu H., Fryar K. L., Landua J., Trevino S. R., Schell D., Thurlkill R. L., Imura S., Scholtz J. M., Gajiwala K. (2014). Contribution of hydrogen bonds to protein stability. Protein Sci..

[2] Scaletti C., Russell P. P. S., Hebel K. J., Rickard M. M., Boob M., Danksagmüller F., Taylor S. A., Pogorelov T. V., Gruebele M. (2024). Hydrogen bonding heterogeneity correlates with protein
folding transition state passage time as revealed by data sonification. Proc. Natl. Acad. Sci. U. S. A..

[3] Juo J., Chen J., Liu P., Hong B., Zhang J., Dong H., Li S. (2023). Microscopic Mechanism of Proton Transfer
in Pure Water under Ambient Conditions. J. Chem.
Theory Comput..

[4] Hassanali A., Giberti F., Cuny J., Kühne T. D., Parrinello M. (2013). Proton transfer through the water
gossamer. Proc. Natl. Acad. Sci. U. S. A..

[5] David R. B., Yaacov A. B., Eren B. (2023). Hydrogen Exchange
through Hydrogen
Bonding between Methanol and Water in the Adsorbed State on Cu(111). J. Phys. Chem. Lett..

[6] Pei A., Xie R., Zhu L., Wu F., Huang Z., Pang Y., Chang Y. C., Chai G., Pao C. W., Gao Q. (2025). Methanol-Enhanced
Low-Cell-Voltage Hydrogen Generation at Industrial-Grade
Current Density by Triadic Active Sites of Pt1–Pdn–(Ni,Co)­(OH)_x_. J. Am. Chem. Soc..

[7] Jansson H., Swenson J. (2011). The slow dielectric Debye relaxation of monoalcohols
in confined geometries. J. Chem. Phys..

[8] Kaatze U. (2017). Dielectric
and structural relaxation in water and some monohydric alcohols. J. Chem. Phys..

[9] Maity N. C., Baksi A., Kumbhakar K., Biswas R. (2023). Impact and structure
of water in aqueous octanol mixtures: Hz-GHz dielectric relaxation
measurements and computer simulations. J. Photochem.
Photobiol. A Chem..

[10] Petong P., Pottel R., Kaatze U. (1999). Dielectric relaxation of H-bonded
liquids. Mixtures of ethanol and n-hexanol at different compositions
and temperatures. J. Phys. Chem. A.

[11] Patil S. P., Chaudhari A. S., Lokhande M. P., Shankarwar A. G., Helambe S. N., Arbad B. R., Mehrotra S. C. (1999). Dielectric Measurements
of Aniline and Alcohol Mixtures at 283, 293, 303, and 313 K Using
the Time Domain Technique. J. Chem. Eng. Data.

[12] Ellison W. J., Lamkaouchi K., Moreau J.-M. (1996). Water: A dielectric reference. J. Mol. Liq..

[13] Buchner R., Barthel J., Stauber J. (1999). The dielectric relaxation
of water
between 0*
^C^
* and 25*
^C^
*. Chem. Phys. Lett..

[14] Barthel J., Bachhuber K., Buchner R., Hetzenauer H. (1990). Dielectric
spectra of some common solvents in the microwave region. Water and
some lower alcohols. Chem. Phys. Lett..

[15] Kao, K. C. Dielectric Phenomena in Solids; Elsevier Academic Press: London, 2004; pp. 92–93.

[16] Coffey W. T. (2004). Dielectric
relaxation: An overview. J. Mol. Liq..

[17] Offei-Danso A., Morzan U. N., Rodriguez A., Hassanali A., Jelic A. (2023). The collective burst mechanism of
angular jumps in liquid water. Nat. Commun..

[18] Priyadarsini A., Dasari S., Mallik B. S. (2020). Thermophysical properties
and angular
jump dynamics of water: A comparative study of DFT and DFT-dispersion-based
molecule dynamics study. J. Phys. Chem. A.

[19] Stirnemann G., Laage D. (2010). Direct evidence of
angular jumps during water reorientation through
two-dimensional infrared anisotropy. J. Phys.
Chem. Lett..

[20] Laage D., Hynes J. (2006). A molecular jump mechanism
of water reorientation. Science.

[21] Arbe A., Malo de Molina P., Alvarez F., Frick B., Colmenero J. (2016). Dielectric
susceptibility in liquid water: Microscopic insights from coherent
and incoherent neutron scattering. Phys. Rev.
Lett..

[22] Gainaru C., Meier R., Schildmann S., Lederle C., Hiller W., Rössler E. A., Böhmer R. (2010). Nuclear-magnetic-resonance measurements
reveal the origin of the Debye process in monohydroxy alcohols. Phys. Rev. Lett..

[23] Gabriel J., Pabst F., Helbling A., Böhmer T., Blochowicz T. (2018). Nature of the Debye-Process in Monohydroxy Alcohols:
5-Methyl-2-Hexanol Investigated by Depolarized Light Scattering and
Dielectric Spectroscopy. Phys. Rev. Lett..

[24] Kampfrath T., Campen R. K., Wolf M., Sajadi M. (2018). The Nature of the Dielectric
Response of Methanol Revealed by the Terahertz Kerr Effect. J. Phys. Chem. Lett..

[25] Weigl P., Koestel D., Pabst F., Gabriel J. P., Walther T., Blochowicz T. (2019). Local dielectric response in 1-propanol:
A-relaxation
versus relaxation of mesoscale structures. Phys.
Chem. Chem. Phys..

[26] Sillrén P., Bielecki J., Mattsson J., Börjesson L., Matic A. (2012). A statistical model of hydrogen bond
networks in liquid alcohols. J. Chem. Phys..

[27] Sillrén P., Matic A., Karlsson M., Koza M., Maccarini M., Fouquet P., Götz M., Bauer T., Gulich R., Lunkenheimer P. (2014). Liquid 1-propanol studied by neutron scattering,
near-infrared, and dielectric spectroscopy. J. Chem. Phys..

[28] Blach S., Forbert H., Marx D. (2025). On the complex
hydrogen-bond network
structural dynamics of liquid methanol: Chains, rings, bifurcations,
and lifetimes. J. Chem. Phys..

[29] Patil S., Sun R., Cheng S., Cheng S. (2023). Molecular mechanism of the Debye
relaxation in monohydroxy alcohols revealed from rheo-dielectric spectroscopy. Phys. Rev. Lett..

[30] Cheng S., Patil S., Cheng S. (2024). Hydrogen Bonding Exchange and Supramolecular
Dynamics of Monohydroxy Alcohols. Phys. Rev.
Lett..

[31] Böhmer R., Gainaru C., Richert R. (2014). Structure
and dynamics of monohydroxy
alcohols— Milestones towards their microscopic understanding,
100 years after Debye. Phys. Rep..

[32] Singh L. P., Richert R. (2012). Watching Hydrogen-Bonded
Structures in an Alcohol Convert
from Rings to Chains. Phys. Rev. Lett..

[33] Soszka N., Hachuła B., Tarnacka M., Kaminska E., Pawlus S., Kaminski K., Paluch M. (2021). Is a Dissociation Process Underlying
the Molecular Origin of the Debye Process in Monohydroxy Alcohols?. J. Phys. Chem. B.

[34] Wieth P., Vogel M. (2014). Dynamical and structural properties
of monohydroxy alcohols exhibiting
a Debye process. J. Chem. Phys..

[35] Johari G. P., Dannhauser W. (1969). Effect of Pressure on Dielectric Relaxation in Isomeric
Octanols. J. Chem. Phys..

[36] Kalinovskaya O. E., Vij J. K. (2000). The exponential
dielectric relaxation dynamics in a
secondary alcohol’s supercooled liquid and glassy states. J. Chem. Phys..

[37] Chua Y. Z., Young-Gonzales A. R., Richert R., Ediger M. D., Schick C. (2017). Dynamics of
supercooled liquid and plastic crystalline ethanol: Dielectric relaxation
and AC nanocalorimetry distinguish structural *α*- and Debye relaxation processes. J. Chem.
Phys..

[38] Ngai K. L., Paluch M. (2004). Classification of secondary
relaxation in glass-formers
based on dynamic properties. J. Chem. Phys..

[39] Minami R., Itoh K., Takahashi H., Higasi K. (1980). A theoretical approach
to the dielectric relaxation of liquid alcohols. J. Chem. Phys..

[40] Kaatze U., Behrends R., Pottel R. (2002). Hydrogen network fluctuations and
dielectric spectrometry of liquids. J. Non-Cryst.
Solids.

[41] Bone R. A., Chung M. K. J., Ponder J. W., Riccardi D., Muzny C., Sundararaman R., Schwarz K. (2024). A new method to calculate broadband
dielectric spectra of solvents from molecular dynamics simulations
demonstrated with polarizable force fields. J. Chem. Phys..

[42] Barthel J., Bachhuber K., Buchner R., Hetzenauer H., Kleebauer M. (1991). A Computer-controlled
System of Transmission Lines
for the Determination of the Complex Permittivity of Lossy Liquids
between 8.5 and 90 GHz. Ber. Bunsenges. Phys.
Chem..

[43] Woodcox M., Mahata A., Hagerstrom A., Stelson A., Muzny C., Sundararaman R., Schwarz K. (2023). Simulating dielectric spectra: A
demonstration of the direct electric field method and a new model
for the nonlinear dielectric response. J. Chem.
Phys..

[44] Ponder J. W., Wu C., Ren P., Pande V. S., Chodera J. D., Schnieders M. J., Haque I., Mobley D. L., Lambrecht D. S., DiStasio R. A. (2010). Current Status of the
AMOEBA Polarizable Force Field. J. Phys. Chem.
B.

[45] Martínez L., Andrade R., Birgin E. G., Martínez J. M. (2009). Packmol:
A package for building initial configurations for molecular dynamics
simulations. J. Comput. Chem..

[46] English N. J., Waldron C. J. (2015). Perspectives on
external electric fields in molecular
simulation: Progress, prospects and challenges. Phys. Chem. Chem. Phys..

[47] Jordan B. P., Sheppard R. J., Szwarnowski S. (1978). The dielectric properties of formamide,
ethanediol and methanol. J. Phys. D: Appl. Phys..

[48] Fukasawa T., Sato T., Watanabe J., Hama Y., Kunz W., Buchner R. (2005). Relation between Dielectric
and Low-Frequency Raman
Spectra of Hydrogen-Bond Liquids. Phys. Rev.
Lett..

[49] Muley P. D., Boldor D. (2013). Investigation of microwave
dielectric properties of
biodiesel components. Bioresour. Technol..

[50] Kumar R., Schmidt J. R., Skinner J. L. (2007). Hydrogen
bonding definitions and
dynamics in liquid water. J. Chem. Phys..

