# Supplementary material for: Chain Formation and Addition Drive the Debye Relaxation of Methanol
Source: J Phys Chem B. 2025 Aug 25;129(35):8946–51. doi: 10.1021/acs.jpcb.5c04122 (PMC12415825; doi:10.1021/acs.jpcb.5c04122)
Supplement: Supplementary file 1 [file jp5c04122_si_001.pdf]

Supplementary Information: Chain Formation and Addition Drive the Debye Relaxation of Methanol

Rebecca A. Bone*
Theiss Research, P.O. Box 127, La Jolla, California, 92038, United States and Material Measurement Laboratory, National Institute of Standards and Technology, 100 Bureau Dr., Gaithersburg, Maryland 20899, United States

Moses K. J. Chung and Jay W. Ponder
Department of Chemistry, Washington University in St. Louis, St. Louis, MO 63130, United States

Kathleen Schwarz†
Material Measurement Laboratory, National Institute of Standards and Technology, 100 Bureau Dr., Gaithersburg, Maryland 20899, United States
(Dated: August 22, 2025)

METHODS

Note: Certain software are identified in this paper to foster understanding. Such identification does not imply recommendation or endorsement by the National Institute of Standards and Technology, nor does it imply that the software identified is necessarily the best available for the purpose. Certain commercial equipment, instruments, or materials are identified in this paper in order to specify the experimental procedure adequately. Such identification is not intended to imply recommendation or endorsement by the National Institute of Standards and Technology, nor is it intended to imply that the materials or equipment identified are necessarily the best available for the purpose.

Code— The input files for our molecular dynamics simulations and the code to perform our configurational analysis can be found in the GitHub repository DielectricSpectroscopyMethanol at https://github.com/usnistgov/DielectricSpectroscopyMethanol.

Spectral calculation and fitting— The imaginary component of the dielectric spectrum was calculated from the total box polarization $\mathbf{P}(t)$ using the Fluctuation Dissipation Theorem using the method developed in 1. The imaginary component of the calculated dielectric spectrum up to a frequency approximately one decade higher than the Debye peak was fit to a single Debye. The static dielectric constant was calculated from the same simulation using the formula:

$$\epsilon_s = \epsilon_\infty + \sum_{\alpha=x,y,z} \frac{\text{var}(P_\alpha)}{3Vk_BT\epsilon_0}, \tag{1}$$

including summing over all three dimensions of the simulation ($\alpha \in x, y, z$). In this calculation, the volume is V, Boltzmann's constant is k_B, the temperature is T, the vacuum permittivity is ϵ_0, and $\text{var}(P_\alpha)$ denotes the variance of the α component of the polarization time series. The high-frequency dielectric constant was determined as in Ref. 1.

Benchmark of dielectric spectrum— The FDT dielectric spectrum we calculated from our Molecular Dynamics (MD) simulations matched the contour of the experimental dielectric spectrum reported in Ref. 2, as shown in Figure S1. Imaginary components at selected frequencies evaluated with the direct electric field method agree well with these values. The static dielectric constant was overestimated in our simulations, but the time constant agreed well as shown in Table S1.

TABLE S1. Fit of Debye peak of experimental and calculated spectrum of methanol.

Spectrum	ϵ_s	$\Delta\epsilon_D$	τ_D (ps)
Simulated	44.04	39.91	45.51
Experimental	32.63	27.217	51.5

* r.a.bone@theissresearch.org
† kathleen.schwarz@nist.gov

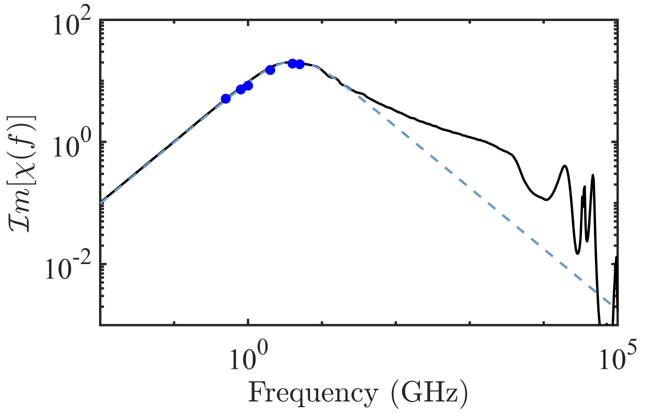

FIG. S1. Imaginary component of the methanol dielectric spectrum calculated using the FDT method (black) and its Debye fit (dashed blue) and a set of points calculated from the direct electric field method (blue markers).

Querying chain events— Where we compared the response before and after certain events in the life cycle of chains, the time scale on which the events were defined was determined by the frequency of the field being applied (see main text). For instance, the amount of time within which a chain may form or die in our definition decreases as the frequency of the field increases. Because we looked for events that change the alignment of molecules in response to the field, we only tracked the amount of change due to an event on the timescale of the field. However, the coarse graining in time led to some unavoidable inaccuracies in the identification of events. To address this, we add the condition that a molecule being considered for these events must not be part of a chain in the time step directly before (in the case of chain formation and addition) or after (in the case of chain death and removal) the event. Thus, we query only the change in orientation associated with going from a lone molecule to a chain or vice versa.

In addition, the time that molecules had to reorient during these events changed with the frequency of the field. At higher frequencies, molecules had less time to reorient during these events, thus resulting in smaller responses at higher frequencies. We queried if this observed response was due to an event in the life of a chain by collecting this response just before and after these events occur. If there was no difference between them, the event was not allowing for reorientation in response to the field.

In Figure S2, the real components of the fit before and after each of the four chain events are not the same. Further, the 95 % confidence intervals of the before and after fits do not overlap at frequency below the Debye peak. This is a strong indication of a change in orientation associated with these events on the timescale of the Debye peak.

We also observe wider error bars in the imaginary fits than in the real fits. This results in overlap in the imaginary fits before and after chain events. There are no trends for any of the life events in the change in the imaginary fit. We will therefore not consider the imaginary fits in our analysis of chain life events.

Angle between specified bond and applied field— For each configuration, we determined the angle that each bond within a molecule in the simulation makes relative to the electric field (along the z-axis). This was done by defining a vector along the axis of the bond,

$$\vec{v}_1 = \{x_2 - x_1, y_2 - y_1, z_2 - z_1\}, \tag{2}$$

and the unit vector of the positive direction of the z-axis,

$$\vec{v}_2 = \{0, 0, 1\}. \tag{3}$$

The angle between these two vectors is given by the formula:

$$\theta(\vec{v}_1, \vec{v}_2) = \arccos\left(\frac{\vec{v}_1 \cdot \vec{v}_2}{||\vec{v}_1|| \cdot ||\vec{v}_2||}\right). \tag{4}$$

The angle from a specific bond in each molecule to the z-axis was thus determined for each molecule in the simulation at each time frame. From here, we averaged as previously described to obtain a time series representing the average angle of a specific bond to the z-axis across a cycle.

This time series of average angle across a representative cycle varies sinusoidally. Using MATLAB's non-linear fitting function, we fit this response to real and imaginary components via the formula:

$$\langle\theta\rangle_{\text{cyc}} = |A|\sin(\omega t) - |B|\cos(\omega t). \tag{5}$$

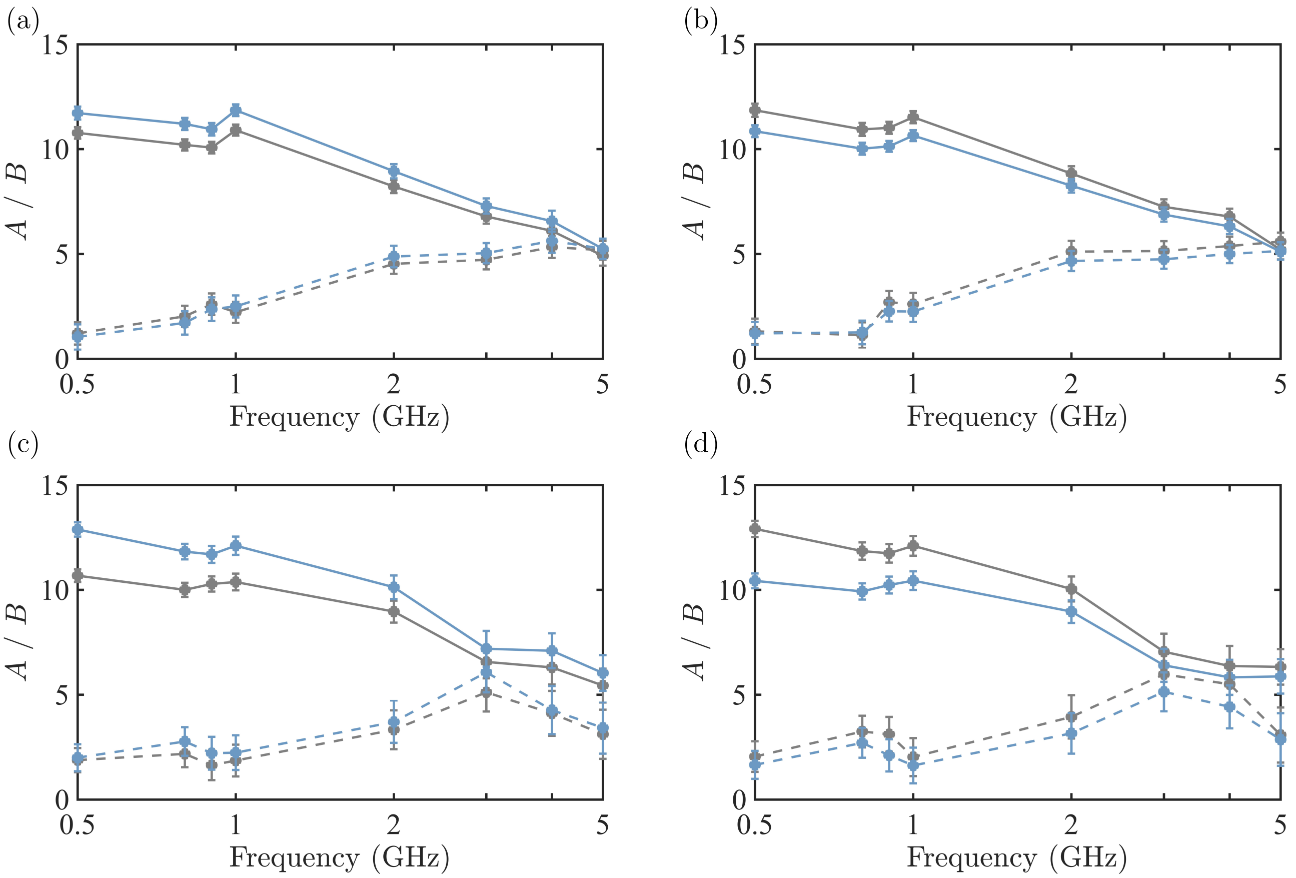

FIG. S2. Real (solid) and imaginary (dashed) fit of the OH orientation before (gray, A_i and B_i) and after (blue, A_f and B_f) (a) chain formation, (b) chain death, (c) addition to chains, (d) removal from chains. Shown averaged over 500,000 chains at field frequencies of 0.5 GHz, 0.8 GHz, 0.9 GHz, 1 GHz, 2 GHz, 3 GHz, 4 GHz, and 5 GHz. 95 % confidence interval shown by error bars.

,with a representative fit illustrated in Figure S3.

Hydrogen bond identification— Our algorithm for identifying hydrogen bonds considered a hydroxyl hydrogen and an oxygen in different molecules within a given radius r_c of each other. Additionally, these atoms formed a hydrogen bond if the O..H-O angle and the H-O..H angle is at least 90°. These requirements ensured that we are looking for hydrogen bonds between hydroxyl groups of separate molecules and that the two hydroxyl groups are in the correct orientation to hydrogen bond. All that remained was the choice of maximal radius r_c.

A too-small r_c failed to identify some of the hydrogen bonds in the MD snapshot. A too-large r_c identified hydrogen bonds incorrectly, including neighbors as hydrogen bonds (as shown in Figure S4).

To illustrate this problem, we generated the distribution of hydrogen bond lengths over an arbitrarily large spatial window. These distributions are shown for methanol and 1-butanol in Fig. S5 (a) and (b) respectively.

The hydrogen bond, neighbor, and second neighbor distributions are superimposed upon each other. We therefore chose for our maximal allowed hydrogen bond distance a value at the minimum between the first and second peaks in the total distribution. This primarily selected for actual hydrogen bonds and discluded the vast majority of neighbor, secondary neighbor, etc. hydrogen bonded molecules from consideration. We found this distance to be 2.6 Å.

Both the atoms and molecules involved in each hydrogen bond were notated. Then, we tracked chains of hydrogen bonded molecules through time.

Algorithm for tracking chains of molecules in time— In order to track chains of molecules through time, we first took the list of extant hydrogen bonds at each time step and determined from that list all chains of hydrogen bonded molecules at that time step only. The molecules involved in that chain and the hydrogen bonds involved in that chain were then logged. We then used a separate piece of code to read the chains that exist at each time step and track them through the simulation. The pseudocode for this is in Algorithm 1.

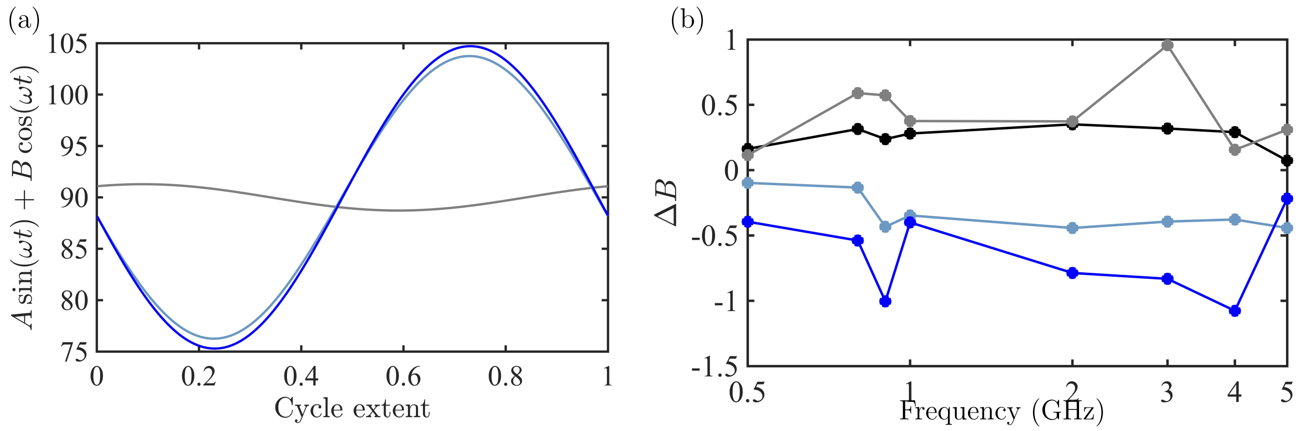

FIG. S3. (a) Fit of pre-addition (light blue) and post-addition (dark blue) angle of the OH bond to the z-axis at 0.5 GHz and without a field (gray). (b) Difference in imaginary part of fit of OH angle to z-axis pre- and post- addition (gray), chain formation (black), chain death (light blue), and removal from chains (dark blue). The error associated with B for chain events is much larger than that associated with A, leading to less reliable trends for the imaginary component shown here.

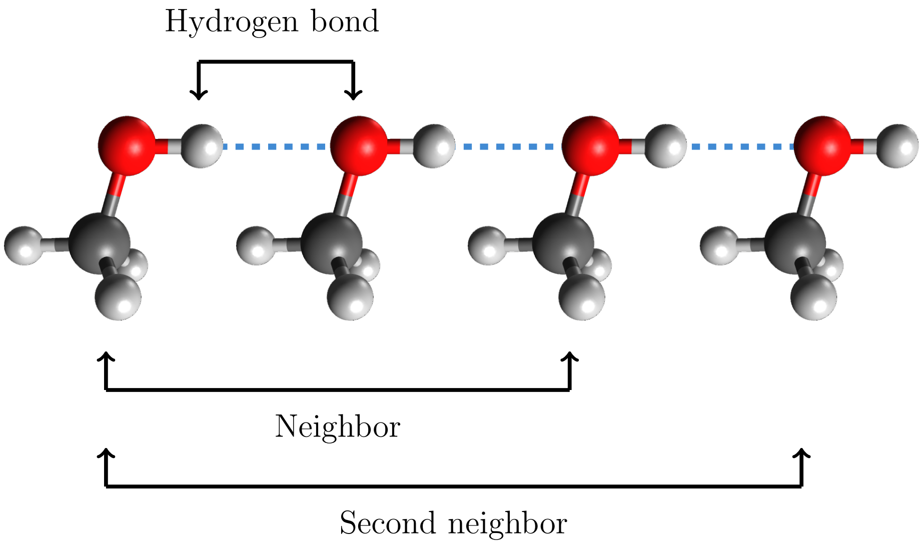

FIG. S4. Diagram of molecules hydrogen bonded in a chain.

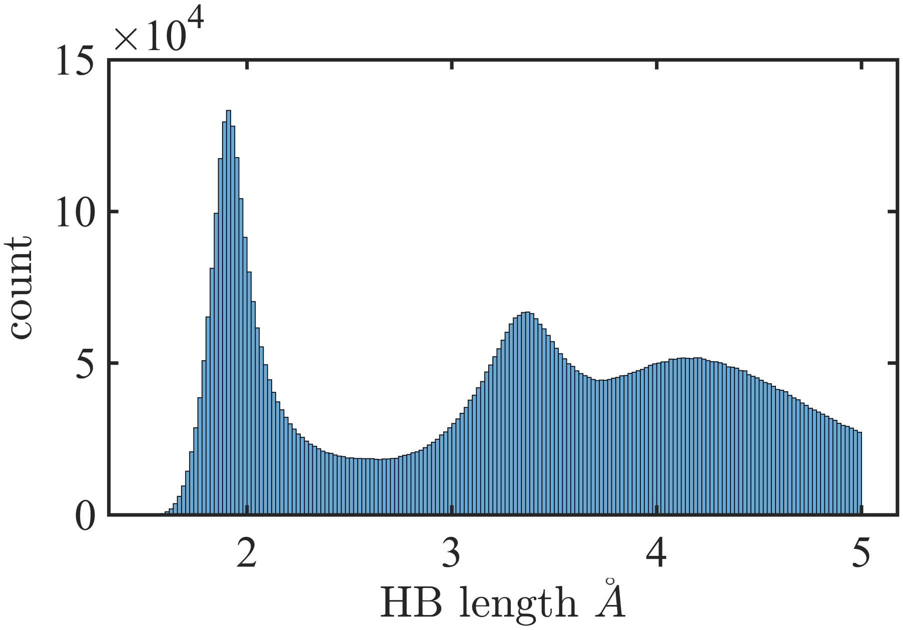

FIG. S5. Distribution of hydrogen bond lengths in methanol.

Algorithm 1 Algorithm for tracking chains of molecules in time

```
Given: simulation of length t_max frames
Initialize complete mol empty list of molecules in completed chains
Initialize complete hb empty list of hydrogen bonds in completed chains
for t in 1..t_max do
    Load in mol list of molecules in each chain
    Load in HB list of hydrogen bonds in each chain
    if t=1 then
        Initialize extant mol list of molecules in each extant chain
        Initialize extant hb list of hydrogen bonds in each extant chain
        for i chains at time t do
            extant mol(i) ← mol(i)
            extant hb(i) ← hb(i)
        end for
    else
        for chains at time t do
            for extant chains at time t-1 do
                Compare list of HB in extant chains to new chains
                if chain(i) at time t is the same as chain(j) at time t-1 then
                    Update listing of extant chain
                    Mark extant chain(j) as completely found
                else if chain(i) contains all of chain(j) then
                    Update listing of extant chain(j) to chain(i)
                    Mark extant chain(j) as completely found
                else if chain(i) contains part of chain(j) then
                    if No other part of chain(j) has yet been found then
                        Update listing of extant chain(j)
                        Mark extant chain(j) as partially found
                    else
                        Create new extant chain with history of chain(j) and current time membership of chain(i)
                    end if
                end if
            end for
        end for
        for extant chains at time t-1 do
            if No part of chain(i) found then
                move extant chain(i) to completed list
            end if
        end for
        for new chains at time t do
            if new chain not found in extant chains then
                Create new extant chain listing
            end if
        end for
    end if
end for
Report all extant chains as completed
```

Dipole moment of hydrogen bound molecules— Once chains of hydrogen bonded molecules were identified and tracked through time, we then determined metrics for each chain over the time that chain exists. Our primary metric here was the dipole moment of the chain. We averaged the dipole moment of chains both over the chains that exist and across cycles of the field. So, the average dipole moment of chains at step i of a cycle was determined from the sum of the dipole moments of all chains that exist at step i of a cycle divided by the number of chains that exist at that step of any cycle. We also determined the total dipole moment due to molecules that are not hydrogen bonded.

Chain and lone molecule lifetime distributions.– Through the analysis detailed above, we can collect starting and ending times of each chain and each lone molecule in a simulation. However, our simulations have various reporting frequencies that depend upon the frequency of the applied field. We leverage these reporting frequencies to find the frequency range, and thus the set of reporting frequencies, at which the distribution of chain lifetimes is converged to a single defined shape with converged parameters. At low frequencies, there are fewer total chances to observe a particular chain. Thus, we expect the distribution at lower reporting frequencies to be less converged than at higher reporting frequencies. Across the frequency range, we observe an exponential shape to the chain lifetime distribution with some over-representation of very short lifetimes due to capturing molecules that are only briefly near each other

rather than truly hydrogen-bonded, as shown in Figure S6. Observation across the range of reporting frequencies then yields a converged rate parameter and thus a converged description of the distribution (still with over-representation of short lifetimes). We take the mean of the converged values to attain a mean chain lifetime of 2.2 ps.

TABLE S2. Mean chain lifetime from fitting of exponential distribution of chain lifetimes at various applied field frequencies separating converged from unconverged values

Frequency (GHz)	Mean chain lifetime (ps)
0.1	19.7
0.5	6.96
0.8	5.22
0.9	4.91
1.0	4.65
2.0	3.30
3.0	2.70
4.0	2.55
5.0	2.64
8.0	2.35
9.0	2.15
10.0	2.30
20.0	2.31
30.0	2.01
40.0	2.31
50.0	2.13

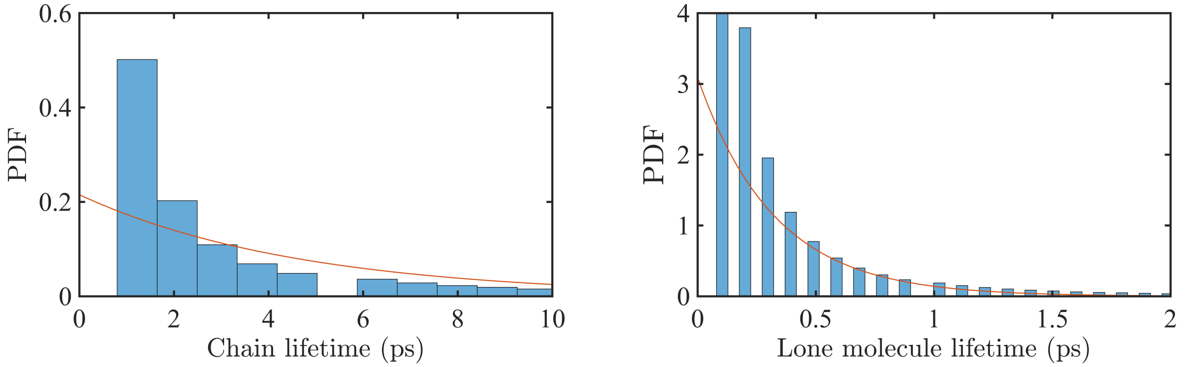

FIG. S6. (a) Histogram of chain lifetimes for 500,000 chains simulated at 1 GHz with exponential fit line shown. (b) Histogram of lone molecule lifetimes from 10 cycles simulated without an electric field and with reporting as though a 10 GHz field had been applied with exponential fit line shown. Note that chains and lone molecules with short lifetimes are overrepresented due to algorithmic choices.

When we use the same approach to get to the lone molecule lifetime distribution, the mean lifetime does not converge. Therefore, we use the lifetime attained from an equilibrium simulation with no applied field at a reporting frequency equal to that used in our simulations with a 10 GHz applied field. When we do this, we find an exponential distribution of lone molecule lifetimes with a mean of 0.3 ps. This functions as an upper bound of the mean lone molecule lifetime.

I. ADDITIONAL MD ANALYSIS

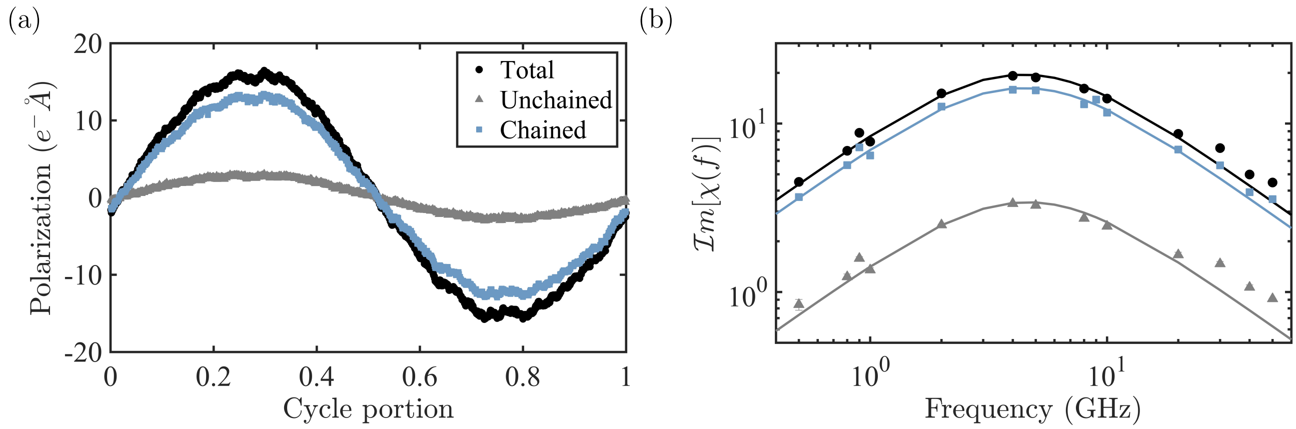

FIG. S7. (a) Total z-component of the polarization of all molecules (black), molecules in chains (blue), and molecules not in chains (gray) across an average cycle. (b) Contribution to the imaginary component of the dielectric spectrum of all molecules (black), molecules in chains (blue), and molecules not in chains (gray). Debye fit of each shown as lines.

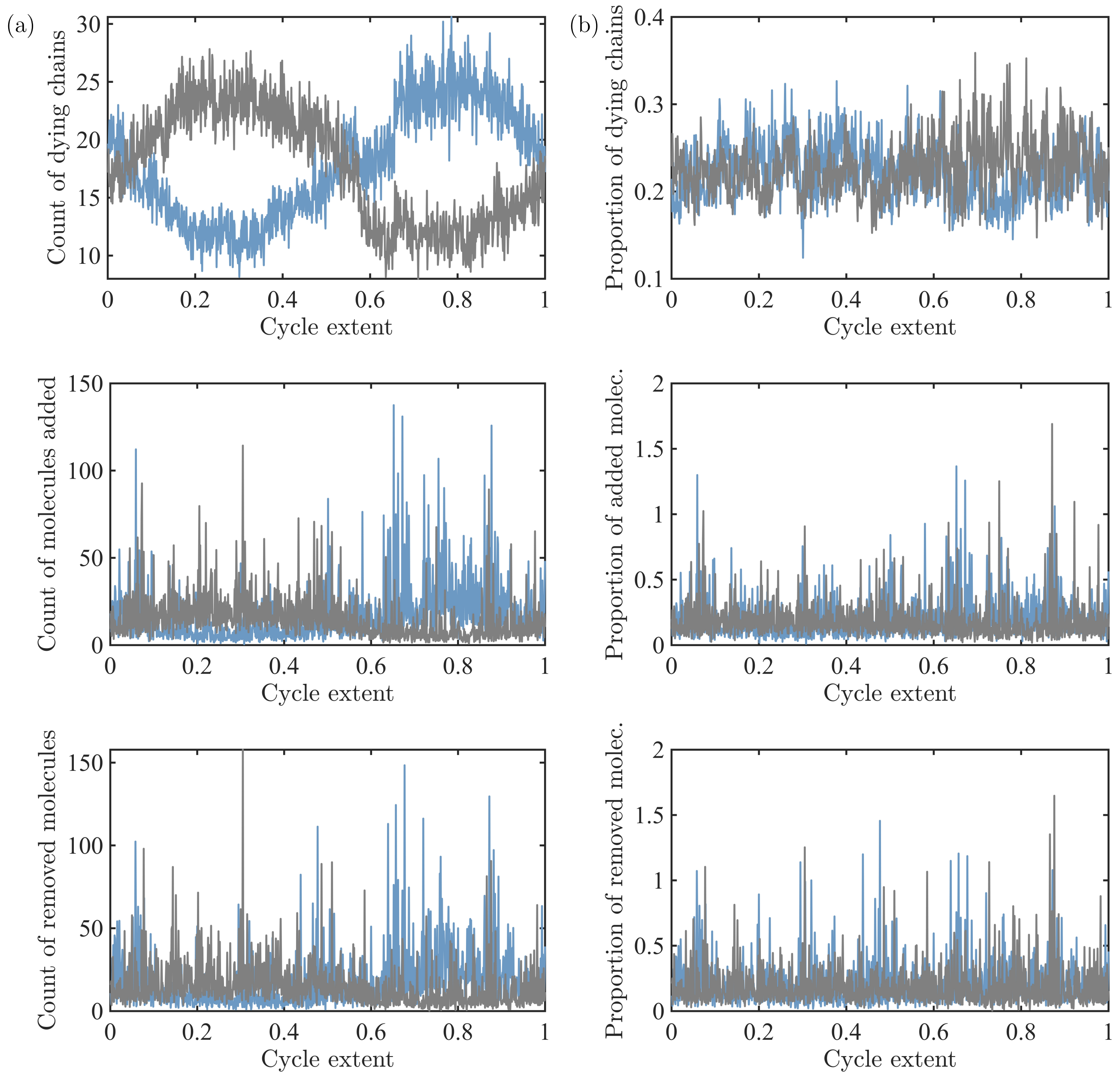

FIG. S8. 1 GHz: Count (a,c,e) and proportion (b,d,e) of (a-b) chains dying, (c-d) molecules added (e-f) molecules removed where the average angle of the OH bond to the z-axis o the chain is greater than 90°(blue) and less than 90°(gray).

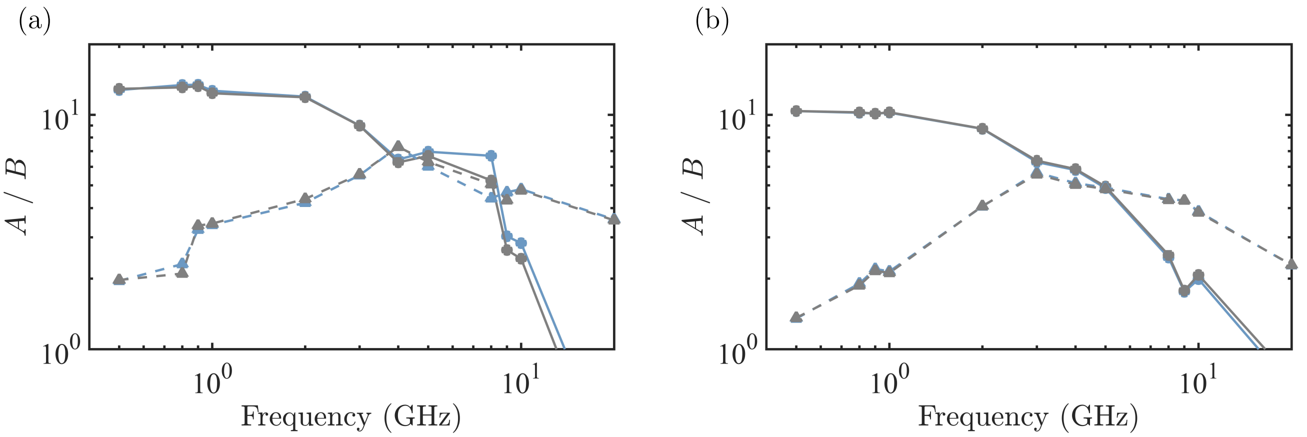

FIG. S9. Real (solid) and imaginary (dashed) fit of the OH reorientation at the start (gray, A_i and B_i) and end (blue, A_f and B_f) of the history of (a) each chain and (b) each lone molecule, averaged as previously described across cycles. In each case, the difference between the fit at the beginning (gray) and end (blue) of the history is not statistically significant (i.e., the points are on top of one another). Thus, there is no statistically significant change in the OH orientation over either the lifetime of chains or the lifetime of lone molecules.

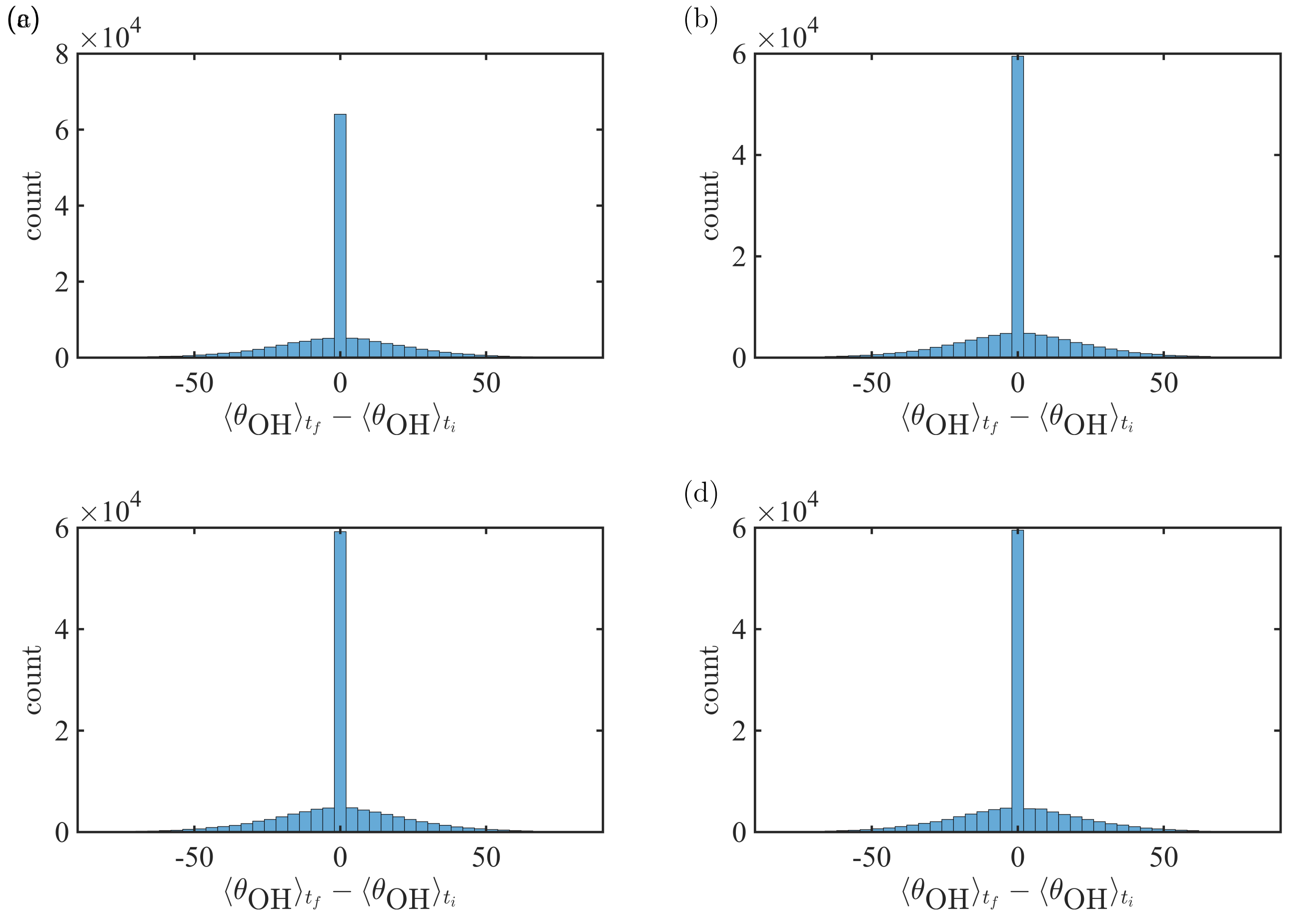

FIG. S10. Histogram of change in average OH bond angle to z-axis across chain lifetime over 500,000 chains starting in (a) first quarter of cycle, (b) second quarter of cycle, (c) third quarter of cycle, (d) fourth quarter of cycle at 0.5 GHz.

[1] Rebecca A. Bone, Moses K. J. Chung, Jay W. Ponder, Demian Riccardi, Chris Muzny, Ravishankar Sundararaman, and Kathleen Schwarz. A new method to calculate broadband dielectric spectra of solvents from molecular dynamics simulations demonstrated with polarizable force fields. *J. Chem. Phys.*, 161:064306, 2024.

[2] J. Barthel, K. Bachhuber, R. Buchner, H. Hetzenauer, and M. Kleebauer. A computer-controlled system of transmission lines for the determination of the complex permittivity of lossy liquids between 8.5 and 90 GHz. *Ber. Bunsenges. Phys. Chem.*, 95:853–859, 1991.
